# Australian recommendations on perioperative use of disease‐modifying anti‐rheumatic drugs in people with inflammatory arthritis undergoing elective surgery

**DOI:** 10.1111/imj.16073

**Published:** 2023-05-22

**Authors:** Rachelle Buchbinder, Vanessa Glennon, Renea V. Johnston, Sue E. Brennan, Chris Fong, Suzie Edward May, Sean O'Neill, Peter Smitham, Lyndal Trevena, Glen Whittaker, Anita Wluka, Samuel L. Whittle

**Affiliations:** ^1^ School of Public Health and Preventive Medicine Monash University Victoria Melbourne Australia; ^2^ Eastern Clinical Research Unit Eastern Health Box Hill Hospital and Monash University Victoria Melbourne Australia; ^3^ Consumer Representative, Giving Voice Western Australia Perth Australia; ^4^ Sydney Musculoskeletal Health Flagship University of Sydney Northern Clinical School and Royal North Shore Hospital New South Wales Sydney Australia; ^5^ Orthopaedic & Trauma Department, Royal Adelaide Hospital & Discipline of Orthopaedics University of Adelaide South Australia Adelaide Australia; ^6^ Faculty of Medicine and Health University of Sydney New South Wales Sydney Australia; ^7^ Discipline of Podiatry, School of Allied Health, Human Services and Sport La Trobe University Victoria Melbourne Australia; ^8^ Rheumatology Unit Queen Elizabeth Hospital South Australia Adelaide Australia

**Keywords:** inflammatory arthritis, perioperative use, disease‐modifying anti‐rheumatic drugs, living guidelines

## Abstract

Disease‐modifying anti‐rheumatic drugs (DMARDs) are effective treatments for inflammatory arthritis but carry an increased risk of infection. For patients undergoing surgery, there is a need to consider the trade‐off between a theoretical increased risk of infection with continuation of DMARDs perioperatively versus an increased risk of disease flare if they are temporarily withheld. We used the Grading of Recommendations Assessment, Development and Evaluation methodology to develop recommendations for perioperative use of DMARDs for people with inflammatory arthritis undergoing elective surgery. The recommendations form part of the National Health and Medical Research Council‐endorsed Australian Living Guideline for the Pharmacological Management of Inflammatory Arthritis. Conditional recommendations were made against routinely discontinuing conventional synthetic and biologic (b) DMARDs in the perioperative period but to consider temporary discontinuation of bDMARDs in individuals with a high risk of infection or where the impact of infection would be severe. A conditional recommendation was made in favour of temporary discontinuation of targeted synthetic DMARDs in the perioperative period.

## Introduction

Disease‐modifying anti‐rheumatic drugs (DMARDs), including conventional synthetic (cs), biologic (b) and targeted synthetic (ts) DMARDs, are the cornerstone of pharmacologic treatment for inflammatory arthritis, including rheumatoid arthritis (RA), psoriatic arthritis (PsA) and axial spondyloarthritis (AxSpA). While they are an effective treatment, they also carry a risk of adverse effects, including an increased risk of infection. This is of relevance for patients undergoing surgery when there is a need to consider the trade‐off between a theoretical increased risk of infection with the continuation of DMARDs through the perioperative period, versus an increased risk of disease flare if these medications are temporarily withheld.^1^ The perioperative period is generally defined as the period around the time of the surgical operation, including preoperative, operative and postoperative stages.

Perioperative use of DMARDs in people with inflammatory arthritis undergoing elective surgery was identified as a priority topic for the Australian Living Guideline through a survey of members of the Australian Rheumatology Association.^2^ This paper presents the newly developed Australian living guidance on this topic.

## Methods

As outlined previously,^3^ the Australian National Health and Medical Research Council (NHMRC)‐endorsed Living Guideline for the Pharmacological Management of Inflammatory Arthritis was established with the aim of helping Australian clinicians and patients keep up with the rapidly evolving evidence base in this field. It employs ‘living evidence’ methodology, in which individual recommendations are updated in near real time as new evidence emerges. All updates to the recommendations, including the addition of new trial evidence, are published immediately through the web‐based application MAGICapp (https://app.magicapp.org/#/guidelines) and can be viewed at www.mskguidelines.org.

Recommendations were developed in accordance with NHMRC guidance,^4^ consistent with evolving living evidence methods,^5^ and using the rigorous Grading of Recommendations Assessment, Development and Evaluation (GRADE) approach.^6^ They were developed from the perspective of the individual patient living with inflammatory arthritis. A detailed description of methods is available at https://app.magicapp.org/#/guideline/LqRV3n/section/EP885E.

## Panel composition and meetings

The recommendations on perioperative use of DMARDs were developed at a meeting held on 24 November 2021. The panel members comprised consumers and clinicians with expertise in rheumatology, orthopaedic surgery, general primary care and allied health. One panel member (CF) was considered at moderate risk of conflict of interest, as adjudicated by a panel of external adjudicators, limiting his contribution to panel discussion only. All other panel members were considered at low risk and therefore contributed to both panel discussion and consensus voting.

## Evidence reviews and development of recommendations

In view of the potential differences between csDMARDs, bDMARDs and tsDMARDs in terms of risk of infection, disease flare and other perioperative adverse events, the panel elected to consider the three medication groups separately.

PICO (Participant, Intervention, Comparison and Outcomes) questions were specified for each class of medication to inform search strategies. Participants included people with RA, PsA and AxSpA. For each question, we collected data from systematic reviews and randomised controlled trials (RCTs) and produced evidence summaries that incorporated a description of all the included studies, summary of findings tables synthesising the findings and the certainty of evidence for each important outcome according to GRADE methodology.^7^

To supplement the evidence base, we also included observational studies comparing perioperative discontinuation versus continuation of any DMARD in adults with inflammatory arthritis undergoing any type of elective surgery. These data were considered as additional evidence in the Evidence to Decision (EtD) process, but not graded or included in the summary of findings tables.

Prior to the guideline meeting, panel members were provided with the evidence summaries and supplementary information. At the meeting, the evidence was reviewed, and the panel was guided through the GRADE EtD framework by a GRADE methodologist (SB); the direction and strength of the recommendation (strong or conditional) were determined by consensus; and wording of each recommendation was formulated. The interpretation of the effects follows GRADE guidance for writing informative statements and incorporates information about the size of the effect and certainty of evidence.^8^ The panel agreed on the interpretation of each result prior to making recommendations.

## Living evidence updates

As outlined previously,^3^ evidence searches underpinning these living recommendations are updated every 3 months. New impactful evidence (e.g. new trial data that alter the benefit‐to‐harm ratio or the certainty of evidence) is rapidly incorporated into an updated evidence summary and presented to the panel and may result in the publication of a new version of a recommendation. The new version may involve changes to the recommendation, its strength and/or supporting text. Users of the recommendations may also provide comments through MAGICapp, which are also used to inform updates to each recommendation.

## Results

### Recommendations

The panel made three separate conditional recommendations (Table 1). They conditionally recommended against routinely discontinuing csDMARDs in the perioperative period. They also conditionally recommended against routinely discontinuing bDMARDs in the perioperative period but to consider temporary discontinuation of bDMARDs in individuals with a high risk of infection or where the impact of infection would be severe. They conditionally recommended consideration of temporary discontinuation of tsDMARDs in the perioperative period.

**Table 1:** Recommendations for the perioperative use of disease‐modifying anti‐rheumatic drugs in people with inflammatory arthritis (rheumatoid arthritis, psoriatic arthritis and axial spondyloarthritis)

Recommendation	Strength of recommendation
Do not routinely discontinue csDMARDs in the perioperative period.	*Conditional recommendation*
Do not routinely discontinue bDMARDs in the perioperative period; consider temporary discontinuation in individuals with a high risk of infection or where the impact of infection would be severe.	*Conditional recommendation*
Consider temporary discontinuation of tsDMARDs in the perioperative period.	*Conditional recommendation*

In the GRADE approach, recommendations are classified as strong or conditional. A strong recommendation means that most people would choose that intervention. A conditional recommendation means that the majority of individuals in this situation would want the recommended course of action, but many would not, and individuals' choices will vary depending on their values and preferences.

bDMARD, biologic disease‐modifying anti‐rheumatic drug; csDMARD, conventional synthetic disease‐modifying anti‐rheumatic drug; GRADE, Grading of Recommendations Assessment, Development and Evaluation; tsDMARD, targeted synthetic disease‐modifying anti‐rheumatic drug.

### Evidence

There were three RCTs, all comparing perioperative discontinuation versus continuation of csDMARDs (methotrexate^9^, ^10^ and leflunomide^11^) in adults with RA undergoing elective orthopaedic surgery. These data were presented to the panel in a single summary of findings table (summarised in Table 2). No trials were identified for perioperative discontinuation versus continuation of csDMARDs for PsA or AxSpA or perioperative discontinuation versus continuation of bDMARDs or tsDMARDs for any type of inflammatory arthritis. No RCTs investigated other types of elective surgery.

**Table 2:** Effect of conventional synthetic disease‐modifying anti‐rheumatoid drug (csDMARD) discontinuation versus continuation in the perioperative period^†^ ^,^ ^‡^

		Absolute effect estimate			
Outcome§	Study results	Continuation of csDMARDs	Perioperative discontinuation of csDMARDs	Certainty of the evidence	Interpretation¶
Flare	Relative risk 32.99 (95% CI 4.54–293.53) Based on data from 224 participants in two trials	0.05 per 1000	1.65 per 1000	Low	Perioperative discontinuation of DMARDs may increase the risk of flare
		Difference: 1.6 more people per 1000 (95% CI 0.18 more to 14.63 more)			
Postoperative infections	Relative risk 1.00 (95% CI 0.31–3.19) Based on data from 306 participants in three trials	31 per 1000	31 per 1000	Low	Perioperative discontinuation of DMARDs may have little or no effect on the number of people with postoperative infections
		Difference: 0 fewer people per 1000 (95% CI 21 fewer to 68 more)			
Prosthetic joint infection	Based on data from 64 participants in one trial	–		Not estimable	One study reported there were no prosthetic joint infections in either group, while two studies did not look at the outcome
Total adverse events	Relative risk 2.05 (95% CI 0.67–6.26) Based on data from 306 participants in three trials^††^	106 per 1000	217 per 1000	Low	Perioperative discontinuation of DMARDs may have little or no effect on the number of people reporting adverse events
		Difference: 111 more people per 1000 (95% CI 35 fewer to 555 more)			
Serious adverse events	Relative risk 1.44 (95% CI 0.48–4.32) Based on data from 242 participants in two trials^‡‡^	47 per 1000	68 per 1000	Low	Perioperative discontinuation of DMARDs may have little or no effect on the number of people reporting serious adverse events
		Difference: 20 more people per 1000 (95% CI 24 fewer to 154 more)			
Mean disease activity score (DAS28)					No studies were found that looked at mean disease activity

Living updates to this evidence table may be found at https://app.magicapp.org/#/guideline/6664.

† The perioperative period is generally defined as the period around the time of the surgical operation, including preoperative, operative and postoperative stages.

‡ No RCTs of csDMARD perioperative discontinuation in psoriatic arthritis or spondyloarthritis and no RCTs of b/tsDMARD perioperative discontinuation for rheumatoid arthritis, psoriatic arthritis or spondyloarthritis were found.

§ The panel rated flare, postoperative infections, prosthetic infection and serious adverse events as ‘critical’ and total adverse events and mean disease activity score as ‘important’.

¶ The interpretation followed GRADE guidance for writing informative statements and incorporated information about the size of the effect and certainty of evidence. The panel agreed on the interpretation of each result prior to making recommendations.

†† Adverse events: Defined as wound morbidity (reddening of wound, discharge from wound), systemic infection or wound dehiscence, loosening of implants or any complication requiring a secondary revision procedure and occurring within 1 year of surgery in Grennan *et al*.: 11/72 (4 reddening of wound, 4 discharge from wound, 1 dehiscence and 2 serious adverse events) in the discontinued group versus 2/88 (1 discharge from wound and 1 dehiscence) in the continued group^9^; Tanaki *et al*. 5/41 (2 infected haematomas, 1 infected necrotic eschar, 1 infected discharge and 1 deep wound abscess) in the discontinued group versus 5/14 (2 infected haematomas, 2 infected necrotic eschars and 1 infected discharge) in the continued group^10^; defined as postoperative infections or delayed wound healing (>15 days) in Sany *et al*.: 6/32 (6 delayed wound healing) in the discontinued group and 4/32 (4 delayed wound healings) in the continued group.^11^

‡‡ Serious adverse events: Defined as loosening of implants or any complication requiring a secondary revision procedure and occurring within 1 year of surgery in Grennan *et al*.: 2/72 in the discontinued group versus 0/88 in the continued group^9^; defined as needing revision surgery in Tanaka *et al*.: 7/41 in the discontinued group versus 6/41 in the continued group^10^; Sany *et al*. reported that no one developed postoperative infections (including prosthetic joint infections).^11^

CI, confidence interval; GRADE, Grading of Recommendations Assessment, Development and Evaluation; RCT, randomised controlled trial.

Based on the RCT data in people with RA, perioperative discontinuation of csDMARDs compared to perioperative continuation of DMARDs may increase the risk of flare and may have little or no effect on the number of people with postoperative infections or the number of people reporting adverse or serious adverse events. The certainty of evidence was rated low due to serious risk of bias and serious imprecision. The panel noted that the participants in the two RCTs investigating methotrexate discontinuation were using lower doses (average 10 mg/week) than are currently used by most patients.^9^, ^10^

There were eight eligible observational studies.^12^, ^13^, ^14^, ^15^, ^16^, ^17^, ^18^, ^19^ Most of the participants had undergone elective orthopaedic surgery (mostly arthroplasty),^12^, ^13^, ^14^, ^15^, ^16^, ^17^ one study also included participants who had undergone other types of surgery,^18^ and one study included participants who had undergone cervical spine surgery.^19^ Four studies investigated perioperative discontinuation of bDMARDs, including TNF inhibitors^12^, ^13^, ^14^ and abatacept,^14^ two studies discontinuation of methotrexate,^16^, ^17^ and two studies discontinuation of combination DMARD therapy^18^ or all DMARDs.^18^ Evidence from these studies largely concurred with the findings from RCTs, indicating there may be an increased risk of flare if DMARDs are discontinued, with no apparent reduction in risk of postoperative infection. The observational data also suggest there may be no reduction in risk of prosthetic joint infection with discontinuation of DMARDs perioperatively.

While the panel voted that there was ‘small net benefit or little difference’ for all DMARDs combined, they noted that the existing RCT evidence applies only to csDMARDs (methotrexate and leflunomide) in people with RA; only observational data were available for bDMARDs and there is an absence of evidence regarding tsDMARDs. They also noted that flares may vary in severity, duration and impact and that there may be various factors that impact on the risk of perioperative flare, including the preoperative disease activity, history of flares, use of combination DMARDs, use of glucocorticoids and the duration of DMARD discontinuation. There are few data to permit accurate estimation of the absolute risk of a perioperative disease flare in the individual patient. The panel also noted that disease flares may be particularly important if they impair successful recovery or rehabilitation from surgery, or if they require additional use of glucocorticoids.

### Evidence to Decision

The panel used the GRADE EtD framework to generate recommendations based on the evidence for benefits and harms and the following considerations.^6^

The GRADE EtD process explicitly considers the importance of health outcomes to those affected by the recommendation and the potential variation between individuals in how they may value these outcomes. We did not identify any qualitative studies that directly investigated values and preferences for this topic. The panel considered it likely that individuals would vary in their weighting of the potential increase in the risk of postoperative infection, and particularly infection of a prosthesis, associated with continuation of DMARDs in the perioperative period, versus the risk of a flare of the underlying inflammatory arthritis associated with interruption of disease‐modifying therapy. The panel noted that flares are known to be associated with an increased long‐term risk of joint damage, disability and cardiovascular disease.^20^, ^21^

Our consumer panellist considered that infection would be a greater concern for many people with inflammatory arthritis, particularly infections that may have severe or irreversible consequences (including those affecting prosthetic joints), but that this would be likely to vary according to the type of surgery, the individual's prior experience (including infections and disease flares) and the potential individual impact of a surgical site infection or other hospital‐acquired infection.

There are no available cost‐effectiveness data regarding the decision to continue or withhold DMARDs in the perioperative period. While temporary interruption of therapy is unlikely to have an important impact on overall costs, major adverse outcomes of surgery in this population, including infection or disease flare, may carry an additional resource burden to the individual patient and to the healthcare system. The panel considered it unlikely that temporary discontinuation would have an important impact on the environmental footprint of DMARDs.

They similarly considered it would be unlikely to have a major impact on health equity, although there may be a disproportionately higher risk where there is limited access to rheumatologist care, and acceptability of the recommendations may vary between different stakeholders. While the recommendation is likely to be feasible to implement, barriers to implementation include variations in the responsibility for perioperative dose adjustment across settings, varying dosing schedules for some DMARDs particularly bDMARDs, and issues such as delayed, rescheduled or urgent surgery.

## Discussion

The panel made conditional recommendations in favour of not routinely discontinuing csDMARDs or bDMARDs in the perioperative period in patients with inflammatory arthritis but to consider temporary discontinuation of bDMARDs in individuals with a high risk of infection or where the impact of infection would be severe. By contrast, the panel made a conditional recommendation in favour of temporary discontinuation of tsDMARDs in the perioperative period. These recommendations apply to all forms of inflammatory arthritis, including RA, PsA and AxSpA.

Table 3 provides practical considerations for how to apply these recommendations within a shared decision‐making framework tailored to an individual patient's circumstances. It is important to emphasise that these recommendations are conditional, being informed by RCT evidence only regarding perioperative discontinuation of csDMARDs in people with RA undergoing elective orthopaedic surgery, observational evidence for bDMARDs and a paucity of evidence regarding tsDMARDs. A conditional recommendation means that most individuals in this situation would want the recommended course of action, but many would not, and individuals' choices will vary depending on their values and preferences.^22^ There are also likely to be differences between drugs within these broad groups, particularly those with different molecular targets and dosing schedules. This means that an individual assessment of the potential risks and benefits of DMARD modification should be made. In addition, the potential risks of infection and, therefore, the balance of potential benefit and harm are likely to differ by the type of surgery that is undertaken (e.g. urgent surgery or interventions that involve incision into the respiratory, alimentary or genitourinary tracts).

**Table 3:** Practical considerations

All decisions regarding the perioperative DMARD regimen in people with inflammatory arthritis should be made within a shared decision‐making framework following a clear discussion of potential benefits and harms, tailored to the individual's circumstances
Consider the potential risks and benefits of temporary discontinuation of DMARDs in the perioperative period based on the following factors: Type and urgency of surgery Risk factors for infection Potential impact of infection or flare
For patients planning to withhold DMARDs perioperatively, consider the following schedule as a guide: For **most bDMARDs**, withhold for one dosing cycle prior to surgery ○ that is, plan surgery at approximately the time of the subsequent dose (e.g. for a monthly injection, aim for surgery in the fifth week after the last injection) For **rituximab**, aim for surgery at least 3 months after the most recent dose For **Janus kinase (JAK) inhibitors**, stop treatment approximately 7 days before surgery For **methotrexate**, withhold for one dosing cycle prior to surgery (i.e. plan surgery 1–2 weeks after the most recent dose) For **leflunomide**, stop treatment approximately 7 days before surgery
Aim to recommence DMARDs when surgical sutures have been removed, adequate wound healing has occurred and there are no other symptoms or signs of infection
Be aware that some b/tsDMARDs (e.g. tocilizumab and JAK inhibitors) may diminish or eliminate the acute phase response, particularly if the drug is discontinued or recommenced close to the time of surgery, and therefore the practitioner should be vigilant for the possibility of infection even in those with normal inflammatory markers.

DMARD, disease‐modifying anti‐rheumatic drug.

While the panel were satisfied that the evidence regarding csDMARDs was sufficient to warrant a conditional recommendation in favour of continuing these drugs without interruption in most individuals undergoing elective surgery, current trial data exist only for patients using methotrexate and leflunomide. While these csDMARDs are those most likely to be considered for transient interruption prior to surgery in patients considered to be at very high risk, the long duration of action of both drugs (particularly leflunomide) is noted. Our conditional recommendation in favour of continuing csDMARDs through the perioperative period for perioperative use of csDMARDs is consistent with other international guidelines.^23^, ^24^

For patients planning to withhold csDMARDS perioperatively, the panel suggested that methotrexate be withheld for one dosing cycle prior to surgery (i.e. plan surgery 1–2 weeks after the most recent dose) and leflunomide be withheld approximately 7 days before surgery. It is unlikely that clinicians or patients would choose to withhold other csDMARDs (sulfasalazine or hydroxychloroquine). In addition, glucocorticoids were not included in this recommendation, although the potential impact of concomitant glucocorticoid use in the perioperative period in people with inflammatory arthritis was noted.

There was a spectrum of opinions within the panel regarding the best approach to bDMARDs. While bDMARDs are potent immunomodulators that are associated with an increased risk of infection in general, the current body of observational evidence does not suggest that continuation of therapy is associated with an important risk of infection. Whether interruption of treatment for an arbitrary period either reduces the risk of infection or has a net beneficial effect versus the risk of disease flare remains unknown. On the other hand, disease flare in the perioperative period is unlikely to be benign, particularly if there is an impact on rehabilitation from surgery or if it results in rescue therapy with glucocorticoids. While the panel also considered a conditional recommendation in favour of temporary discontinuation for most patients (except those low risk of infection or where treatment interruption may unnecessarily delay surgery), ultimately, they reached consensus in favour of a conditional recommendation to continue bDMARDs in most people other than those at higher risk of infection or its consequences.

If a decision is made to withhold bDMARDS perioperatively, for most bDMARDs, the panel recommended withholding medication for one dosing cycle prior to surgery, that is, plan surgery at approximately the time of the subsequent dose (e.g. for a monthly injection, aim for surgery in the fifth week after the last injection); for rituximab, aim for surgery at least 3 months after the most recent dose.

While both the 2022 American College of Rheumatology (ACR)/American Association of Hip and Knee Surgeons (AAHKS)^24^ and 2019 British Society of Rheumatology guidelines (up to date as of 23 March 2022)^25^ recommend withholding bDMARDs prior to surgery, both indicate this should be balanced against the perioperative risk of flare, particularly in individuals whose disease has been difficult to control. Of note, the consumer panel participating in the 2017 ACR/AAHKS guidelines^26^ considered the risk of infection to be much more important than the risk of flare, and this was noted to be a strong driver of that guideline's cautious approach to the use of both bDMARDs and tsDMARDs in the perioperative period. On the other hand, a study performed in the United Kingdom that included focus groups with patients, rheumatologists and orthopaedic surgeons reported that patients prioritised avoidance of disease flare.^1^

The panel were also divided about the best approach to tsDMARDs as disease flares may be more common with interruption of tsDMARD therapy. Given the current lack of evidence regarding this class of medications, the potential impact on the risk of infection and other perioperative complications, including the possibility of blunting of the acute phase response in the setting of infection and a theoretical increase in the risk of thrombosis,^27^ the panel agreed on a conditional recommendation to temporarily discontinue tsDMARDs perioperatively. They acknowledged that the optimal timing is unknown but noted that withholding tsDMARDs from approximately 1 week before surgery is likely to be a reasonable approach to balancing the risk of infection versus disease flare.

The conditional recommendation to withhold tsDMARDs in the perioperative period is consistent with other international guidelines.^24^, ^25^ However, the 2022 ACR/AAHKS Guideline, published subsequent to our panel meeting, updated the advice about how long tsDMARDs should be withheld prior to surgery from 1 week to only 3 days.^24^ This updated guidance was made based upon a substudy of a randomised trial comparing continuation versus temporary discontinuation of tofacitinib that demonstrated a rapid increase in disease activity in the latter group.^28^ However, the guideline also noted that a longer period might be considered in patients at higher risk of infection and that this recommendation did not take into account the thrombotic risk potentially associated with this class of drug.

In the absence of evidence to inform the timing of the reinstitution of therapy, the panel considered it reasonable to recommence therapy when surgical sutures have been removed, adequate wound healing has occurred and there are no other symptoms or signs of infection. For most people, this is likely to be 1–2 weeks following surgery. Finally, it was noted that some DMARDs, particularly those that target the IL‐6 pathway (e.g. tocilizumab), and possibly JAK inhibitors, may dampen the acute phase response, and therefore clinicians should remain vigilant to the possibility of infection even in the presence of normal inflammatory markers.

The current recommendations highlight important evidence gaps, particularly regarding the use of b/tsDMARDs in the perioperative period, indicating a need for further research on this topic. These recommendations will be continuously updated over time as new evidence becomes available, and the updated versions will be freely available online through MAGICapp. A recommendation regarding perioperative use of glucocorticoids will be made at a future time.
